# Segregation analysis comparing liability and quantitative trait models for hypertension using the Genetic Analysis Workshop 13 simulated data

**DOI:** 10.1186/1471-2156-4-S1-S79

**Published:** 2003-12-31

**Authors:** GP Crockford, DT Bishop, JH Barrett

**Affiliations:** 1 Genetic Epidemiology Division, Cancer Research UK Clinical Centre in Leeds, Cancer Genetics Building, St James's University Hospital, Beckett Street, Leeds, United Kingdom

## Abstract

Discrete (qualitative) data segregation analysis may be performed assuming the liability model, which involves an underlying normally distributed quantitative phenotype. The appropriateness of the liability model for complex traits is unclear. The Genetic Analysis Workshop 13 simulated data provides measures on systolic blood pressure, a highly complex trait, which may be dichotomized into a discrete trait (hypertension). We perform segregation analysis under the liability model of hypertensive status as a qualitative trait and compare this with results using systolic blood pressure as a quantitative trait (without prior knowledge at that stage of the true underlying simulation model) using 1050 pedigrees ascertained from four replicates on the basis of at least one affected member. Both analyses identify models with major genes and polygenic components to explain the family aggregation of systolic blood pressure. Neither of the methods estimates the true parameters well (as the true model is considerably more complicated than those considered for the analysis), but both identified the most complicated model evaluated as the preferred model. Segregation analysis of complex diseases using relatively simple models is unlikely to provide accurate parameter estimates but is able to indicate major gene and/or polygenic components in familial aggregation of complex diseases.

## Background

Liability models for the segregation analysis of discrete traits assume an underlying normally distributed quantitative phenotype [1]. For relatively simple disease models this method performs well in terms of identifying the underlying genetic models [2-4]. The appropriateness of the liability model for complex diseases is less clear; few substantial studies have included a measured underlying quantitative trait with which discrete data can be naturally compared. The Genetic Analysis Workshop 13 simulated data provide measures on a quantitative trait, systolic blood pressure (*sbp*), which may also be dichotomized to form a discrete trait (hypertension). This is a suitably large and realistic set of data on which comparisons of quantitative and discrete trait models may be made.

The aims of this study were to use both quantitative and qualitative phenotypes in segregation analysis (blind to the simulated model) and compare results with respect to 1) best fitting models (one or two major genes, polygenic and mixed models) and 2) parameter estimates. After initial analysis, the simulated model was examined and found to be highly complex, possibly too complex for segregation analyses to determine whether gene(s) segregate with the trait. Height was chosen as an alternative trait, based on a simpler genetic model within the simulation, as a comparison.

## Methods

### Phenotypic data

For each replicate the phenotypic data for Cohorts 1 and 2 were merged with the pedigree data to form families, the pedigree structures of which are the same for each replicate, and the complete data set was used (no missing data). The variables selected are *famid*, *id*, *dadid*, *momid*, *sex *merged with phenotypic data in Cohort 1 *age *(time-points 1–21), *exam at death*, *hypertensive treatment *(1–21), *sbp *(1–21), *height *(1,5,10,13–21) and in Cohort 2 *age *(1–5), *exam at death*,*hypertensive treatment *(1–5), *sbp *(1–5) and *height *(1–5). For each individual the exam used in the analysis was randomly selected from the first exam through to the last exam or the exam prior to the exam at death, thereby simulating the ascertainment of individuals with a range of histories and ages.

For the quantitative trait, if treatment starts prior to the selected exam, then *sbp *was taken as the last pre-treatment *sbp *and *age *is taken as the corresponding age (using the complete data at least one pre-treatment *sbp *was always available). The distribution of the quantitative trait was positively skewed, but after log-transformation it was approximately normally distributed (*lnsbp*). *Sbp *is age dependent. Adjusting for age for *sbp *removes 14% of the variance. The residual *lnsbp *is the focus of the analysis.

The *sbp *recorded in exams up to and including the randomly selected exam were used to determine the qualitative trait, hypertensive status. Individuals are regarded as affected if they have *sbp *> 140 mm Hg, or receive treatment for high blood pressure, at any of these exams, and the earliest such exam gives the age at disease onset. Current age is age at the randomly selected exam.

Examination of the simulation model for *sbp *revealed a complex mode of inheritance. In order to compare results for a simpler trait, height was also analyzed. Height was adjusted for sex ([height - sex specific mean height]/sex-specific standard deviation in height), and where height is not recorded in the exam selected (first cohort only) the sex-adjusted height from the exam closest to the selected one is used.

### Segregation analysis

Segregation analysis was performed on 1050 families ascertained from four randomly selected replicates (055, 079, 093 and 097). Families were ascertained on the basis of having at least one affected member (*sbp *> 140 or on treatment for hypertension). In families with only one affected member, this individual was designated as the proband. In families with more than one affected individual, a proband was randomly selected from among the affected individuals in the family. Ascertainment correction was performed on all families using the designated probands.

Quantitative trait models were fitted allowing for no familial effects (sporadic), a single major gene (dominant, recessive, or codominant), two major genes, polygenes only, or major genes plus polygenic effects (mixed models). The models allowing for a major gene assume that the phenotype is normally distributed within each genotype, with mean varying according to genotype but equal within-genotype variances.

Variance-components analysis is used to estimate heritability. Random mating and Hardy-Weinberg equilibrium are assumed.

Qualitative trait analysis mirroring the quantitative analyses was performed using a liability model. This model assumes a higher liability threshold needs to be exceeded for younger age at onset. The analysis was carried out with age-specific incidence rates constraining affection probabilities. Age- and sex-specific incidence rates were calculated from all individuals from both cohorts in the full set of 100 replicates, using age at last exam for those still alive and unaffected, age at exam prior to death for those who died, or age at first exam with either *sbp *> 140 or treatment for hypertension for those affected. Analyses using age- and sex-specific incidence data showed very similar parameter estimates for males and females, so for simplicity we report here the results of analyses based on combined incidence data entered as 18 liability classes of 5-year intervals 1–5, 6–10, 11–16, ..., 81–85, and 86–90.

Quantitative trait analysis for sex-adjusted height using all pedigrees in the four replicates was performed using similar models to those for *sbp*. In addition, a three-allele single locus model was considered, since the most important gene in the model underlying the simulation had three alleles.

Analyses were carried out using PAPv5 [5]. Maximizations were determined from several starting values and those at boundary values were scrutinized further to confirm as far as possible that the true maxima were achieved. Tabulated results report differences in twice the log likelihood for each fitted model compared to the baseline sporadic model. Nested models are compared using the likelihood ratio test and non-nested models using the Akaike information criterion [6].

## Results

The 1050 pedigrees ascertained for at least one person with hypertension range in size from 7 family members to 84 with 50% of the pedigrees having 12 or fewer members and 90% having 26 or fewer members. There are only 27 pedigrees with 40 or more members. Families with only the proband affected account for 28%, while those with 3 or more affected account for 50%. Families with 8 or more cases account for 5% of all families, the maximum number affected being 21. Systolic blood pressure was measured on 9776 family members aged 20 to 88.

Quantitative trait analysis in Table 1 shows that the model with two major genes plus polygenes is the best fitting model of those evaluated. For this model the two major genes are codominant and the within-genotype heritability is 41%.

For the qualitative trait analysis the best fitting model of those evaluated is again the mixed model with two major genes. The parameter estimates define a rare recessive and a dominant locus (Table 2). Each of the models including polygenic effects maximize on or near a boundary value (heritability of 1.0). Subsequent analyses confirm (as far as possible) that global maxima have been reached. The program did not converge to a global maximum when trying to fit the single-gene codominant mixed model: the model reported in Table 2, with dominance 0.99, was found by fixing all other parameters.

Modelling height as a simpler trait using the same four replicates with no ascertainment selection, 11,346 individuals had data available on height. The best fitting model for sex-adjusted height is a mixed two-locus model and is significantly better than any single-locus, pure polygenic, or two-locus model (Table 3). The model estimates heritability to be 48%, a codominant (dominance 0.52) locus with allele frequency 0.4 and a recessive (dominance 0.26) locus with allele frequency 0.3. These are reasonable estimates of the true parameters for the first two major genes (disease allele frequencies each 0.3 with codominant and recessive transmission), which account for 40% and 20% of the variance. This leaves 40% of the variance unexplained, and according to the underlying model a further 24% of the total variance is explained by genes. This equates to a residual heritability of 60% (24/40), consistent with the estimate of 48% from the model.

Although a three-allele single-locus mixed model has a better likelihood than any single-locus model, it is costly in terms of the number of parameters required (13 estimated for genotypic means and standard deviations and heritability, plus 3 fixed for known allele frequencies). The likelihood of this model is not significantly better than the two-locus model but the estimate of heritability is comparable (55% estimated when 73% expected based on the residual heritability in the true model).

## Discussion

Both the quantitative and qualitative analyses identified the most complex models considered (two-locus mixed models) as the preferred models, but the model parameter estimates are quite different from each other. In neither case do the parameter estimates match those of the major genes that explain the majority of the genetic variation in the underlying model. All models fitted are much simpler than the highly complex "true" model for *sbp*. Comparisons modeling the simpler quantitative trait of height worked better than *sbp *and retrieved reasonably accurate parameter estimates.

The effect of age is allowed for in the models either by adjusting for age (in the quantitative analysis) or using age-specific incidence data (qualitative analysis) to constrain affection probabilities. It is possible that these constraints may be the source of different parameter estimates for the two methods and in particular heritability estimates of 1.0 in qualitative analyses. Parameter estimates are more closely aligned between the two methods when simpler models without incidence data and age adjustment were investigated (data not shown).

The underlying model for *sbp *includes baseline genes, slope genes, and genetic effects mediated through other factors such as height, weight, and smoking status. The complexity of the model may therefore be such that one- or two-locus segregation analyses will be unable to give a good indication of mode of inheritance. The simulated data provided information on height as a simpler trait, based on 10 baseline genes of which 3 account for most of the sex-specific variation. Our analyses, although limited by the extra complexity of the three-allele locus in the true model, found that the two-locus mixed models provided reasonable estimates of the allele frequencies and dominance of the first two loci and of residual heritability. A three-allele single locus mixed model using the known allele frequencies was not preferred to the best fitting two-locus mixed model. Using two alleles when modeling complex traits with more than one locus seems a reasonable compromise in terms of model accuracy when there are unknown parameters.

The complexity of the simulated model (and possibly reality) inevitably means that simple models will be unable to provide reliable parameter estimates, but such models may indicate the likely components of inheritance. As such, these methods may be useful in studying complex diseases when one or more causative genes have already been identified in combined linkage-segregation analyses.

## Figures and Tables

**Table 1:** Parameter estimates for quantitative trait segregation analyses of age-adjusted *sbp*

Model	Allele frequency	Dominance	Displacement	Heritability	2lnL^A^	Number of parameters
Sporadic	(-)	(-)	(-)	(0)	0	0
Polygenic	(-)	(-)	(-)	0.78	2203.63	1
Single genes						
Dominant	0.30	(1)	1.71	(0)	1237.16	2
Recessive	0.73	(0)	1.76	(0)	1248.84	2
Codominant	0.51	0.51	3.39	(0)	1899.80	3
Mixed models						
Dominant	0.23	(1)	0.00	0.78	2203.63	3
Recessive	0.13	(0)	1.74	0.80	2230.07	3
Codominant	0.13	0.07	1.85	0.80	2230.74	4
2-locus model						
Locus 1	0.56	0.56	3.88			
Locus 2	0.22	0.32	3.81	(0)	2279.30	6
2-locus mixed model						
Locus 1	0.53	0.59	3.20			
Locus 2	0.19	0.26	3.31	0.41	2339.49	7

^A^Twice log likelihood difference from sporadic model. Parameters in parentheses fixed.

**Table 2:** Parameter estimates from qualitative trait segregation analyses of hypertension (including incidence data)

Model	Allele frequency	Dominance	Displacement	Heritability	2lnL^A^	Number of parameters
Sporadic	(-)	(-)	(-)	(0)	0	0
Polygenic	(-)	(-)	(-)	1.00	3623.09	1
Single genes						
Dominant	0.05	(1)	2.98	(0)	3208.07	2
Recessive	0.33	(0)	3.47	(0)	3130.10	2
Codominant	0.07	0.66	4.63	(0)	3421.01	3
Mixed models						
Dominant	0.21	(1)	77.4	1.00	3848.22	3
Recessive	0.73	(0)	5.51	1.00	3779.37	3
Codominant	0.20	0.99	76.9	1.00	3988.55	4
2-locus model						
Locus 1	0.15	0.76	2.94			
Locus 2	0.07	0.75	6.10	(0)	3620.38	6
2-locus mixed model						
Locus 1	0.07	0.02	3.08			
Locus 2	0.18	0.99	102.41	1.00	4002.82	7

^A ^Twice log likelihood difference from sporadic model. Parameters in parentheses fixed. Maximizations at parameter boundaries (heritability 1.0) were further evaluated confirming that the true maximum was attained.

**Table 3:** Parameter estimates for quantitative trait segregation analyses of sex-adjusted height

Model	Allele frequency	Dominance	Displacement	Heritability	2lnL^a^	Number of parameters
Sporadic	(-)	(-)	(-)	(0)	0	0
Polygenic	(-)	(-)	(-)	0.77	2607.39	1
Single genes						
Codominant	0.39	0.45	3.58	(0)	2394.36	3
Mixed models						
Codominant	0.35	0.34	2.53	0.64	2810.17	4
2-locus models						
Locus 1	0.53	0.49	3.25			
Locus 2	0.34	0.35	3.19	(0)	2792.91	6
2-locus mixed model						
Locus 1	0.38	0.52	2.61			
Locus 2	0.33	0.26	2.92	0.48	2842.52	7

^A ^Twice log likelihood difference from sporadic model. Parameters in parentheses fixed. For a single locus model with three alleles at fixed frequencies (0.4, 0.3, and 0.3), 12 parameters were estimated (genotypic means and standard deviations) which maximize at 2lnL = 2750.89; with the additional heritability parameter (13 parameters estimated) 2lnL = 2849.08 and h^2 ^= 0.552.
